# A Genetic Screen in *Drosophila* Reveals Novel Cytoprotective Functions of the Autophagy-Lysosome Pathway

**DOI:** 10.1371/journal.pone.0006068

**Published:** 2009-06-29

**Authors:** Andrew M. Arsham, Thomas P. Neufeld

**Affiliations:** Department of Genetics, Cell Biology, and Development, University of Minnesota, Minneapolis, Minnesota, United States of America; University of Texas MD Anderson Cancer Center, United States of America

## Abstract

The highly conserved autophagy-lysosome pathway is the primary mechanism for breakdown and recycling of macromolecular and organellar cargo in the eukaryotic cell. Autophagy has recently been implicated in protection against cancer, neurodegeneration, and infection, and interest is increasing in additional roles of autophagy in human health, disease, and aging. To search for novel cytoprotective features of this pathway, we carried out a genetic mosaic screen for mutations causing increased lysosomal and/or autophagic activity in the *Drosophila melanogaster* larval fat body. By combining *Drosophila* genetics with live-cell imaging of the fluorescent dye LysoTracker Red and fixed-cell imaging of autophagy-specific fluorescent protein markers, the screen was designed to identify essential metazoan genes whose disruption causes increased flux through the autophagy-lysosome pathway. The screen identified a large number of genes associated with the protein synthesis and ER-secretory pathways (*e.g.* aminoacyl tRNA synthetases, *Oligosaccharyl transferase*, *Sec61α*), and with mitochondrial function and dynamics (*e.g. Rieske iron-sulfur protein, Dynamin-related protein 1*). We also observed that increased lysosomal and autophagic activity were consistently associated with decreased cell size. Our work demonstrates that disruption of the synthesis, transport, folding, or glycosylation of ER-targeted proteins at any of multiple steps leads to autophagy induction. In addition to illuminating cytoprotective features of autophagy in response to cellular damage, this screen establishes a genetic methodology for investigating cell biological phenotypes in live cells, in the context of viable wild type organisms.

## Introduction

The proteasome and the lysosome are the two major routes for cellular digestion of macromolecules. The ubiquitin-proteasome system is highly regulated, specific, and energy-intensive, and biophysical constraints limit the proteasome to degradation of individual proteins (reviewed in [1]). In contrast, the lysosome, an acidic membrane-bound organelle containing a broad spectrum of acid hydrolases, degrades substrates non-specifically, and direct regulation of individual steps is minimal. The large size of lysosomes allows degradation not only of individual proteins, but of large complexes, lipids, and whole organelles, and enables recycling of the resulting raw materials (reviewed in [2]). Defects in lysosomal function cause upwards of 40 lysosomal storage disorders that result in a buildup of undigested material and a wide spectrum of often organ- or cell type-specific secondary effects including neuronal damage or degeneration, and mild to severe developmental impairment (reviewed in [3]). Bulk cargo for degradation is delivered to the lysosome by macroautophagy, a cellular recycling process conserved from yeast to man (hereafter referred to simply as autophagy). Autophagy enables cellular survival in nutrient-poor environments, removal of old or damaged organelles and macromolecules, and is implicated as a protective factor in a wide variety of human disease, from cancer to neurodegeneration to pathogen defense (reviewed in [4]).

Initially characterized genetically and biochemically in yeast [5], autophagy is a multi-step process. The “core” machinery of autophagy involves nearly 20 proteins, and much progress has been made in understanding such unique features as ubiquitin-like conjugation reactions involving both proteins and lipids, and the formation and enlargement of the unusual double membrane enclosure [2]. Fusion of the autophagosome to the lysosome releases the cargo-containing inner membrane into the lysosome for degradation by the more than 50 resident acid hydrolases. After degradation, effluxors mediate the release of nutrients to the cytoplasm for reuse [6], a key step in autophagy's primal function as a response to nutrient deprivation.

A high degree of homology between yeast and metazoans has enabled the study of the autophagy-lysosome pathway in multicellular animals. In both yeast and metazoans, target of rapamycin (TOR) signaling suppresses autophagy in nutrient-replete environments as part of its growth-promoting function, and permits autophagy when cells are starved. In higher organisms, TOR not only responds to nutrients but is also a major effector of growth factor signaling through the PI3K pathway, thus tying autophagy to the regulation of cell growth and cancer [7] and to the coordination of organismal growth and feeding behavior (reviewed in [8]).

Autophagy is also regulated by developmental signals. In *Drosophila*, autophagy is induced during the late larval stages by the steroid hormone ecdysone [9], allowing the animal to break down and recycle larval material in the course of metamorphosis. While autophagy is not strictly required for embryonic development in flies [10] or mice [11], [12], it appears to be necessary for embryonic implantation [13] and perinatal survival in mice [12]. Loss of autophagy also impairs T- and B-cell development, proliferation, and function [14], [15]. Autophagy declines with age, and this decline has long been thought to play a role in aging-related cellular damage and senescence; in support of a role for autophagy in aging, it has recently been shown to be required (though not sufficient) for dietary restriction-induced lifespan extension in worms [16], [17]. Autophagy thus functions not only in cell autonomous responses to nutrient depletion as in yeast, but in regulatory and disease processes unique to multicellular organisms.

Because the bulk of our current genetic and biochemical knowledge of autophagy comes from seminal early genetic screens in yeast, it is not clear to what degree newer functions of autophagy rely on metazoan-specific signaling pathways. We therefore designed a genetic screen to search for novel cytoprotective features of the autophagy-lysosome pathway by looking for mutations that activate the pathway as a signaling or defense response. Using existing genetic tools in *Drosophila* we screened mitotic mosaic clones homozygous for lethal P-element insertions that caused enlargement of the lysosomal compartment as measured by LysoTracker Red, a fluorescent dye. Experiments were performed using collections of FRT-linked recombinogenic P-element insertions on *Drosophila* chromosome 2 L, and were carried out in the larval fat body, the primary nutrient storage organ of the developing larva, and one in which background lysosomal and autophagic activity are extremely low.

The screen revealed a large number of gene disruptions which amplify LysoTracker activity (which we termed LT+), typically in concert with decreased cell and nuclear size, and which were enriched for genes involved in protein synthesis, folding, transport, and degradation, and those involved in mitochondrial function and morphology. The former group included tRNA charging, translocation into the ER, glycosylation, and the unfolded protein response (UPR), while the latter included electron transport and mitochondrial fusion and fission. Because of the large size and monolayer tissue architecture of fat body cells and the sophisticated recombination and gene expression tools generated by the *Drosophila* community, the mosaic approach in fat body allows for the visualization of cell biological phenotypes in live cells in the context of a fully developed wild type animal organ and provides a useful set of tools for investigating a wide range of cellular phenotypes including cell size and shape, intracellular trafficking, and organellar dynamics.

## Results

### A Genetic Screen Reveals a Large Number Of Mutants With Elevated and Punctate LysoTracker Staining

To increase the odds of identifying novel regulators of lysosomal trafficking and autophagy, we designed a screen to identify mutations that activate or derepress, as opposed to block, lysosomal accumulation. We thus expected the screen to identify genes whose activity normally suppresses lysosomal accumulation or autophagy, or genes whose mutations cause cellular stress or damage that would induce these pathways as a defense response. We investigated essential genes using publicly available collections of lethal P-element insertions linked to centromere-proximal FRT recombination sites generated by the Szeged Stock Center and the BruinFly project [18]. We focused our analysis on 383 recessive-lethal P-element insertions on chromosome 2 L (∼20% of the genome), representing a substantial fraction of the predicted essential loci on this chromosome arm.

**Figure 1:**
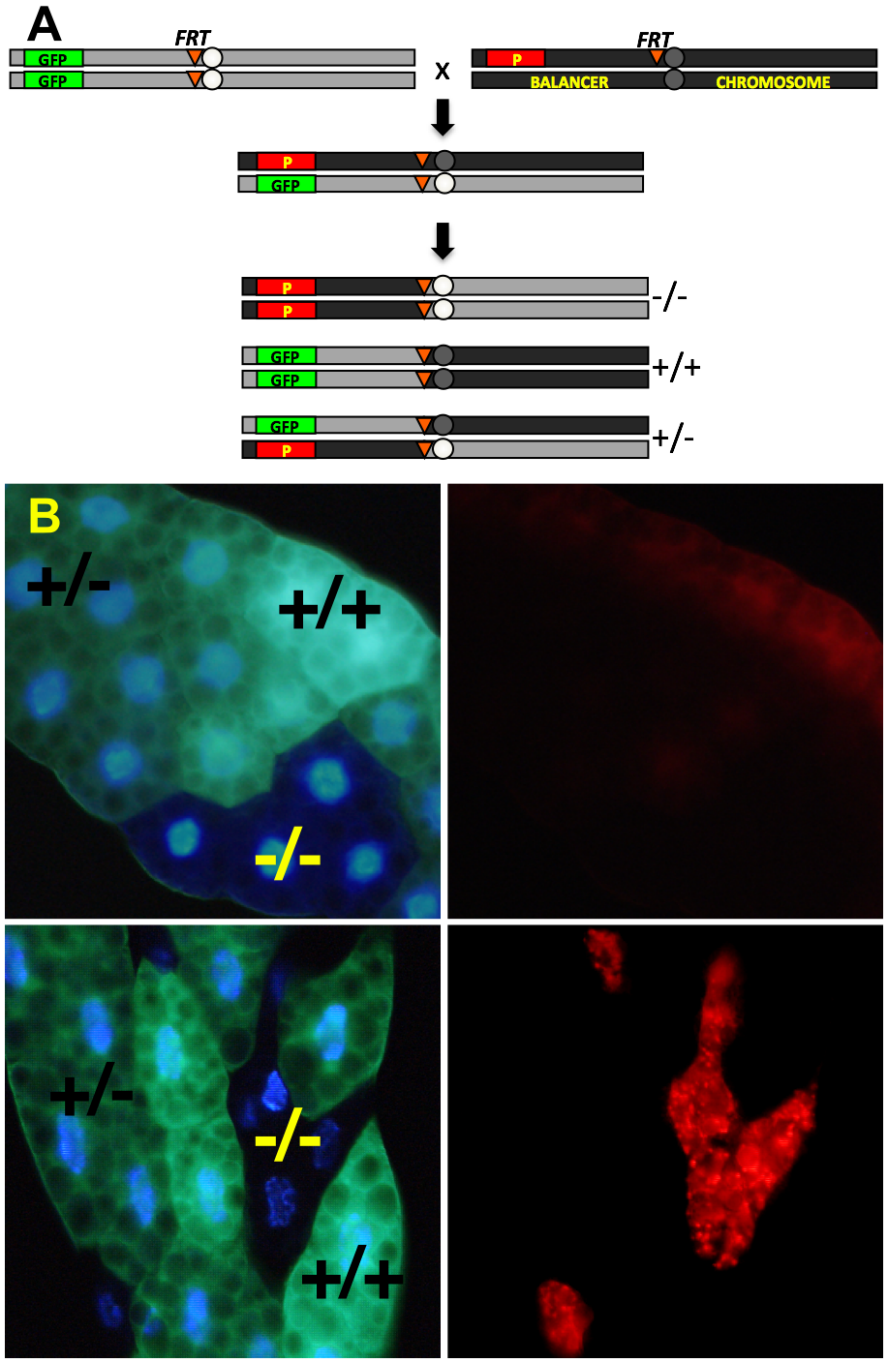
System of mitotic recombination for screening increased lysosomal activity in mosaic mutant *Drosophila* fat body. Adult virgin females of the GFP indicator strain (*y w hsflp*; *UAS-2xeGFP FRT40A Fb-Gal4*, hereafter referred to as FRT40AGFP) were crossed to males carrying P-element-induced mutations distal to FRT40A on chromosome 2 L (A). 6–8 hr embryo collections were immediately heat shocked at 37°C for 1 hour to activate the Flp recombinase. Flp-catalyzed mitotic recombination and subsequent chromosomal segregation early in embryogenesis leads to three cellular genotypes, distinguishable in the larval fat body by their GFP dosage: mutant (−/−, 0 GFP); heterozygote (+/−, 1 GFP); and wild type (+/+, 2 GFP). (B) The top panels show a negative control cross between FRT40AGFP and an isogenized FRT40A wild type strain, with LysoTracker staining on the right. Bottom panels show mutant clones with markedly increased LysoTracker staining relative to neighboring internal control cells, a phenotype which we designate LT+. All panels are unfixed dissected tissue imaged epifluorescently.

The FLP-FRT recombination system was used to generate homozygous mutant mitotic clones for each of the 383 P insertions. Mutant clones were identifiable by their lack of GFP expression and proximity to homozygous twin-spots which had 2 copies of the GFP gene, in otherwise normal heterozygous animals (Fig. 1). The genetic dosage of GFP was easily distinguishable under an epifluorescent microscope. Mosaic fat bodies from well-fed early third instar F1 larvae were dissected and immediately stained with LysoTracker Red, a fluorescent dye which is taken up by negatively charged membrane-bound cellular compartments including autolysosomes and lysosomes. This enabled simple and rapid assessment of the level of lysosomal activity in mutant cells vs. wild type cells in a single microscopic field. As shown in Figure 1, the very low baseline level of LysoTracker staining in the fat bodies of well-fed wild type animals allowed easy identification of cells with elevated LysoTracker staining (denoted LT+) versus normal (LT−).

Table 1 shows the number of LT+ and LT− mutants in the two P-element collections and an EMS-mutagenized population used to pilot the genetic and cell biological techniques used in the screen. The table also shows the number of mutants that displayed reduced size but no discernable LysoTracker phenotype. LT+ mutants comprised 9% and 18%, respectively, of the Szeged and BruinFly collections, as compared to 4% of the EMS mutants. This enrichment compared to the EMS set is not unexpected, given that the P-elements were pre-selected to have recessive lethal phenotypes, but suggests that even when using EMS, there are a large number of mutable genes affecting lysosomal trafficking pathways. Nearly all LT+ mutants also had reduced cell size of varying severity, but the converse was not true—some mutations led to small cells with no elevation in LysoTracker staining (7% and 3% of the Szeged and BruinFly collections; Table 1). In no case did we observe dominant or non-autonomous induction of LysoTracker staining in heterozygous cells. We also identified a number of cell lethal mutants that generated wild type twin spots but no apparent corresponding mutant clone, implying that recombination had occurred but that the mutant cells did not survive. The difference in LT+ mutants between the two sets of P-elements is balanced by a higher rate of LT− small cell phenotypes as well as apparent cell lethals in the Szeged vs. the BruinFly set. Both sets of flies had very similar numbers of LT− mutants.

**Table 1:** Number and percentage of mutants with LysoTracker, cell size, or cell lethal phenotypes.

	LT−	LT+	Small cells & LT−	Cell lethal	Total
Szeged	88 (77%)	10 (9%)	8 (7%)	8 (7%)	114
BruinFly	201 (75%)	48 (18%)	7 (3%)	13 (4.8%)	269
EMS	450 (95%)	21 (4%)	n/a	n/a	471

### Gene Ontology Analysis Identifies Functional Categories Enriched Among Lysotracker Positive Mutants

**Figure 2:**
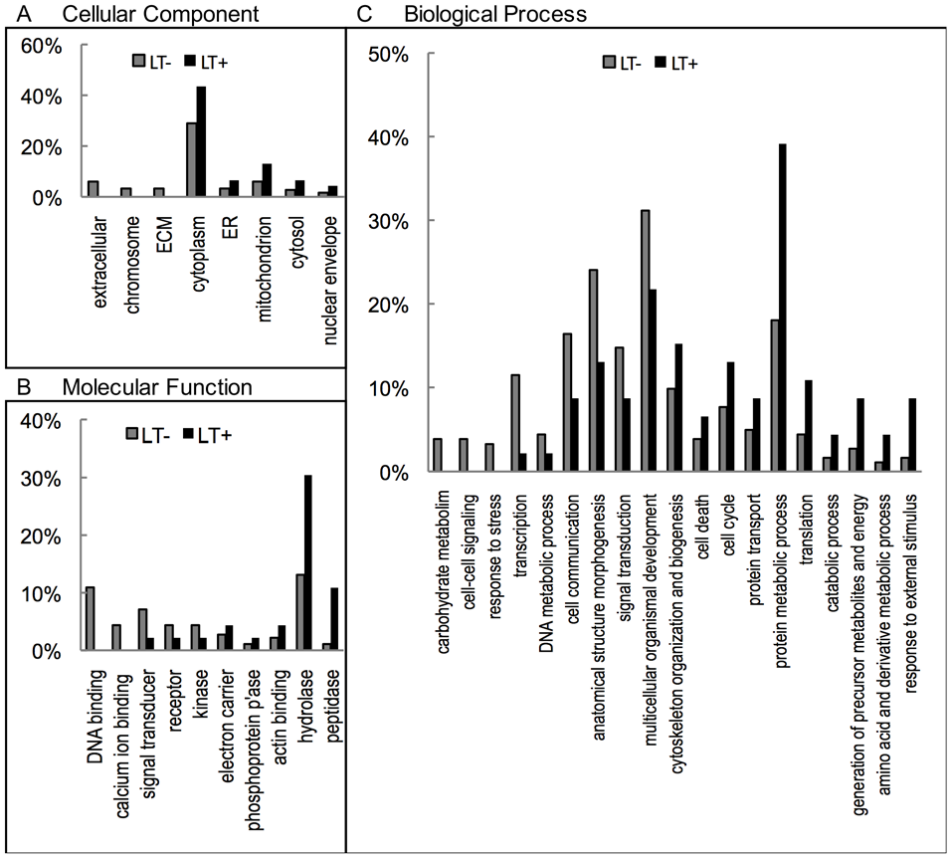
Gene ontology categories for LT+ and LT− mutants. Annotated genes associated with P-element insertions were assigned upper level gene ontology terms by the GO Term Mapper online software. The percentage of LT+ and LT− genes in selected categories are shown for cellular component (A), molecular function (B), and biological process (C). Not every gene is assigned GO terms, some genes have multiple GO terms, and categories are not mutually exclusive and therefore do not add up to 100%.

Gene Ontology (GO) terms for all mutants were assigned and compared between LT+ and LT− groups (Fig. 2; Table S1 shows all GO assignments), to look for enrichment of particular functional categories. GO terms are assigned from three independent groupings: cellular component, biological process, and molecular function. Figure 2 shows selected GO categories from all three groupings for which notable differences between phenotypic groups were found—the histogram shows the percent of each group that is assigned to a certain GO term. Of 57 LT+ candidate genes, 46 were assigned GO terms. The rest were not present in the GO database, suggesting that they had yet to be categorized. It should be noted that GO terms are not mutually exclusive. Because proteins can have multiple functional categories, the frequencies do not add up to 100%. Of particular interest were enrichments within the LT+ group in the cellular components ER and mitochondria (1.99 and 2.17 fold enrichment, respectively, Fig. 2A); the molecular functions electron carrier activity, actin binding, and peptidase activity (1.59, 1.99, and 9.95 fold, respectively, Fig. 2B); and the biological processes translation, catabolic process, generation of precursor metabolites and energy, and amino acid and derivative metabolic process (2.49, 2.65, 3.18, and 3.98 fold, respectively, Fig. 2C). Because of the small sample size for many of these categories and statistical correction for the large number of comparisons, the increased association of mutations in specific functional categories with LysoTracker activation, while suggestive, is not statistically significant and should be interpreted with caution. In addition, many of the GO assignments are made by computerized algorithms on the basis of sequence or structural homology to known proteins or domains, which presents the possibility of both false positive and false negative results. GO terms are nonetheless helpful in discerning patterns and trends within and between large groups of genes.

### Disruption of tRNA Synthetases Increases Lysosomal Activity

**Figure 3:**
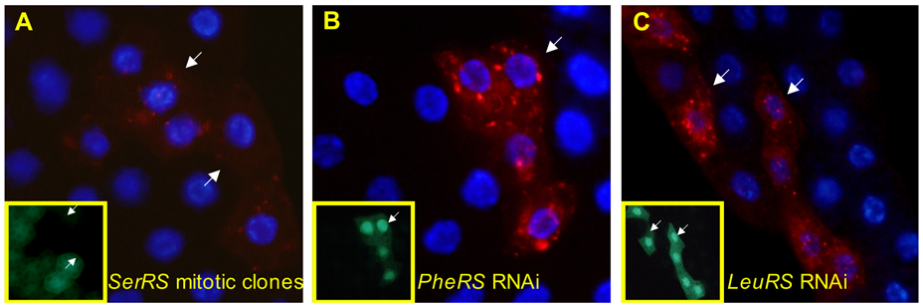
Disruption of tRNA synthetase genes increases LysoTracker staining. (A) Clones of cells homozygous for P-element insertion KG03126 in the seryl tRNA synthetase *CG17259* (indicated by white arrows and by lack of GFP in the inset panel) display punctate LysoTracker staining under fed conditions. (B and C) Cell clones expressing UAS-driven RNAi (indicated by GFP expression in inset panels) against phenylanalyl (B) and leucyl tRNA synthetases (C) display punctate LysoTracker staining. Allele used for mitotic clones: *CG17259^KG03126^_._*

Aminoacyl tRNA synthetases (AARS) catalyze the loading of tRNAs with their cognate amino acid. P-element insertion in the 5′ UTR of the gene *CG17259*, the seryl-tRNA synthetase (*SerRS*), caused elevated LysoTracker staining (Fig. 3A), while a P-element insertion in the first intron of threonyl-tRNA synthetase (*ThrRS*, FlyBase symbol *Aats-thr*) showed weak and intermittent LT+ phenotypes (data not shown). To extend these results, we used a clonal RNAi approach to inhibit expression of a panel of AARS genes. Briefly, stochastic FLP-FRT recombination events cause the looping out of a transcription stop signal embedded in a GAL4 expression construct driven by the constitutive Act5c promoter. The resulting clonal expression of GAL4 then activates transcription of stably integrated RNAi and GFP expression constructs driven by similar GAL4-responsive promoters. This system enables the analysis of RNAi effects on small numbers of GFP-marked cells in otherwise healthy organisms.

RNAi directed against a variety of AARSs (phenalanyl, leucyl (Fig. 3B–C), and prolyl, tyrosyl, methionyl, and tryptophanyl (data not shown)) was expressed, and larval fat bodies were stained with LysoTracker as above. Cells expressing RNAi against any of these genes had reduced cell and nuclear size and increased punctate LysoTracker phenotype. That a wide range of tRNA synthetases of both major subclasses (class I and II) displayed this phenotype strongly suggests that this result is broadly applicable to all tRNA synthetases irrespective of target codon or enzyme structure. Interestingly, we saw no evidence of increased autophagosome formation in these cells (data not shown), indicating that the LT+ phenotypes may reflect increased flux through endocytic rather than autophagic pathways.

### Disruption of Mitochondria-Associated Genes Increases Lysosomal and Autophagic Activity

Autophagic transport to lysosomes is the major route for degradation of organelles, and mitochondria in particular have been a subject of intensive study within the autophagy field. 13% (6/46) of all categorized LT+ genes in our collection are classified as mitochondrial, compared to just 6% in the LT− group and 4.3% in the entire genome. In addition, *Dynamin-related protein 1 (Drp1)* and *abrupt (ab)* are not recognized as mitochondrial in the gene ontology system but have nonetheless been experimentally shown to play a role in mitochondrial dynamics, indicating that GO term mapping may underestimate the number of mitochondria-associated genes.

**Figure 4:**
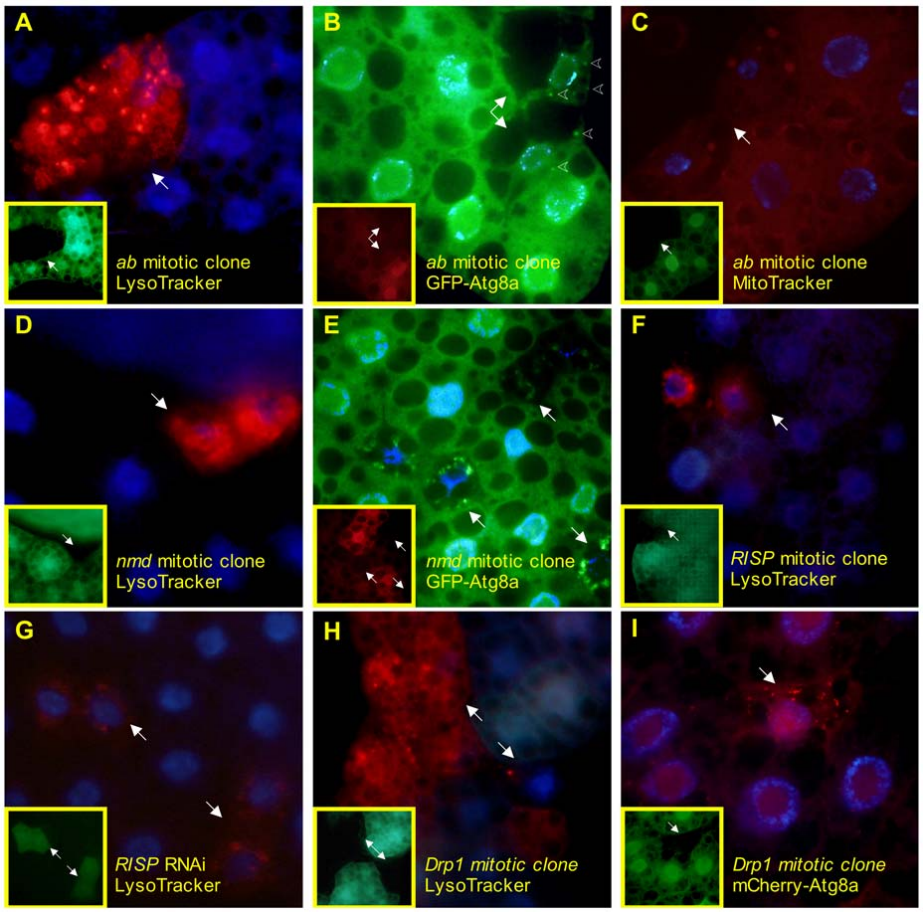
Disruption of mitochondria-associated genes leads to increased LysoTracker and autophagy. Clones of cells homozygous for P-element insertions in *ab*, *nmd*, *RISP*, and *Drp1* display punctate LysoTracker staining (A, D, F, H, unfixed tissue). *Ab*, *nmd*, and *Drp1* mutations also lead to redistribution of fluorescent Atg8a into autophagosomes (B, E, I, fixed tissue, mutant clones are indicated here and elsewhere by solid white arrows; representative Atg8a punctae in B are indicated by arrowheads). Cells homozygous for the *nmd* insertion also show aberrant mitochondrial morphology as visualized by MitoTracker Red dye (C, fixed tissue) compared to neighboring cells. Cells expressing RNAi against *RISP* (indicated by GFP expression, G, unfixed tissue) have punctate and perinuclear LysoTracker staining. Inset panels indicate mutant clones which lack GFP or RFP, or Gal4-activated cells which express GFP, as appropriate. Alleles used for mitotic clones: *ab^k02807^, nmd^k10909^, RFeSP^k11704^, Drp1^KG03815^.*

Selected results are shown in Figure 4, including P-element insertions in *ab, Drp1, no mitochondrial derivative* (*nmd*), and *Rieske iron sulfur protein* (*RISP*; FlyBase symbol *RFeSP*, Fig. 4A, D, F, H). While all 4 mutants induce a typical LT+ phenotype, *RISP* disruption additionally leads to an unusually perinuclear concentration of LysoTracker punctae, which was replicated by expression of RNAi against *RISP* (Fig. 4G). To discern whether the increased lysosomal volume correlated with an increase in autophagy, we expressed either GFP or mCherry fusion proteins of Atg8a/LC3, an essential autophagy protein which localizes to autophagosomal membranes—punctate subcellular localization of fluorescent Atg8 fusions is commonly used as an indicator of active autophagy. Atg8a-labeled vesicles can also accumulate due to a block in autophagy at a post-induction step, but the dual observation of both Atg8a and LysoTracker punctae strongly supports an increase in the overall autophagic flux. *Ab*, *nmd*, and *Drp1* mutation all caused formation of Atg8a punctae (Figs. 4B, E, I). *Ab* mutant cells also had reduced overall GFP-Atg8a levels compared to neighboring wild type cells, suggesting that some mutations may lead to enhanced autophagosome-lysosome fusion and increased autophagic flux, causing a high rate of turnover of the fusion protein. To ascertain if the mutations in these mitochondria-associated genes caused obvious differences in the volume or the spatial distribution of mitochondria, we visualized mitochondria with MitoTracker Red. While most of the mutants did not show obvious differences in mitochondrial phenotypes, *ab* caused mitochondria to aggregate in large clumps (Fig. 4C) in mutant cells. This is consistent with recent results suggesting that *ab* plays a role in mitochondrial sorting and that mutations in this gene cause a Parkinson's-like neurodegenerative syndrome in flies (R. Cox and A. Spradling, personal communication).

### Disruption of the ER Translocon, SRP, and other Regulators of Protein Folding and Trafficking Increase Both Autophagy and Lysosomal Volume

**Figure 5:**
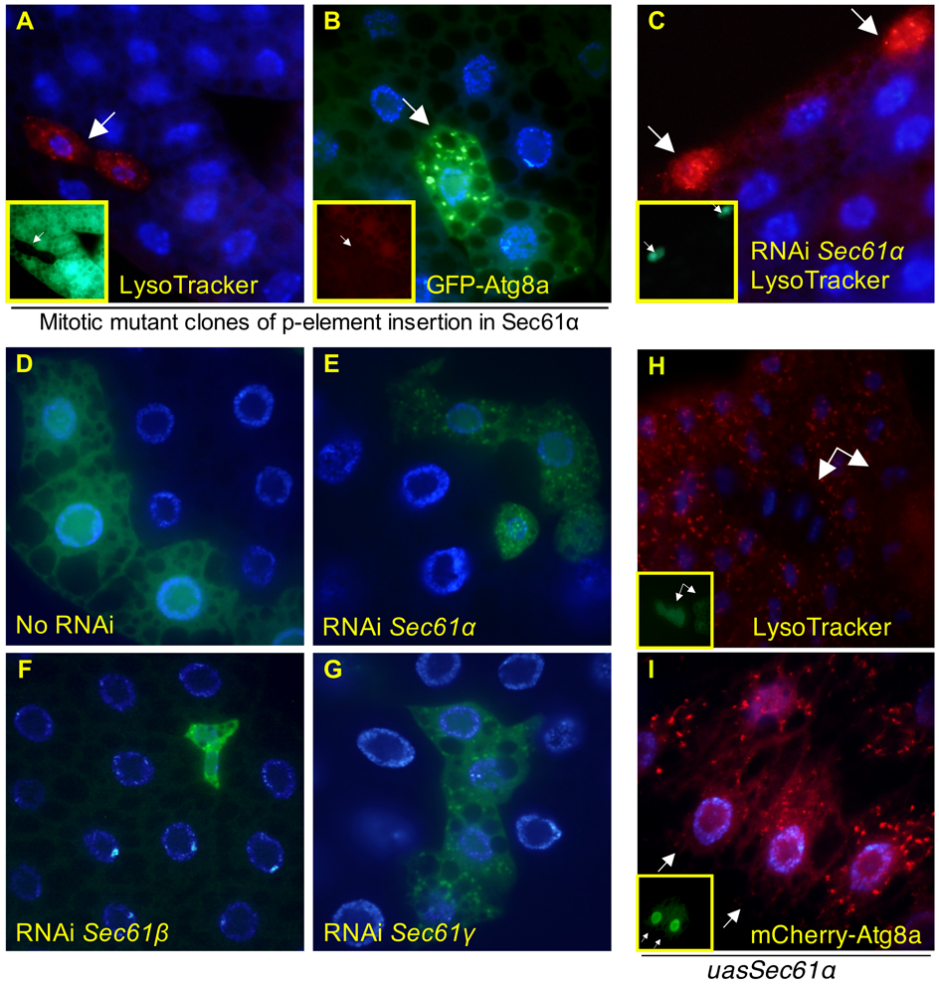
Disruption of the ER translocon induces autophagy. Cells homozygous for P-element insertions in *Sec61α* display elevated and punctate LysoTracker (A) and GFP-Atg8a (B). Flies expressing RNAi against *Sec61α* also have elevated and punctate LysoTracker (C). (D–G) Flies expressing RNAi against *Sec61α*(Ε), *Sec61β*Φ, *Sec61γ*(Γ), have increased numbers of autophagosomes, compared to the no-RNAi control (D). Cells with elevated *Sec61α* suppress starvation-induced autophagy as measured by LysoTracker dye (H) and mCherry-Atg8a fusion protein (I). (A, C, H, unfixed tissue; B, D, E, F, G, I fixed tissue. Inset panels and arrows indicate mutant clones lacking GFP or RFP, or Gal4-activated cells which express GFP, as appropriate.) Allele used for mitotic clones: *Sec61α^k04917^*.

Cells homozygous for a P-element insertion in the *Sec61α* gene had markedly elevated and punctate LysoTracker staining as well as punctate localization of GFP-Atg8a (Figs. 5A, B). RNAi against the *Sec61α* gene similarly led to punctate patterns of LysoTracker (Fig. 5C) and GFP-Atg8a (Fig. 5E). Sec61α is part of a trimeric complex, and having confirmed that disruption of the gene by both P-element and RNAi leads to elevated autophagy, we next sought to test the effects of disruption of the two other subunits, Sec61β and Sec61γ. RNAi targeted against *Sec61β* (Fig. 5F) or *γ* (Fig. 5G) induced LysoTracker staining (data not shown) and redistribution of GFP-Atg8a into a punctate pattern. Conversely, overexpression of ectopic *Sec61α* suppressed starvation-induced LysoTracker staining (Fig. 5H), and resulted in fewer and smaller mCherry-Atg8a-containing autophagosomes (Fig. 5I). Given that the ER translocon is a trimer, extra Sec61α would not be expected to increase the ER's overall protein transport capacity, suggesting that Sec61α may be playing a regulatory role in autophagy in addition to its presumed transport role.

**Figure 6:**
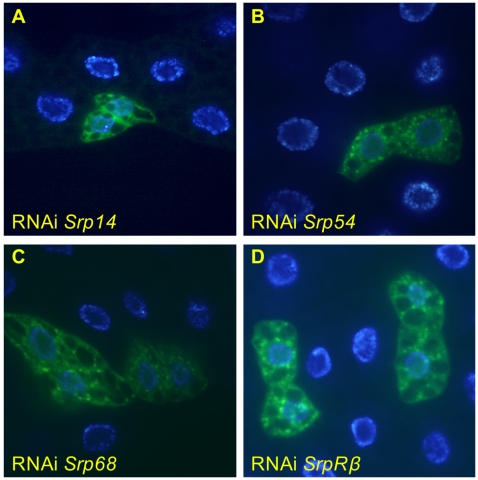
Disruption of the signal recognition particle and its receptor induce autophagosome formation. Cells expressing inducible RNAi against the indicated subunits of the SRP and SRP receptor, marked by GFP-Atg8a expression, have elevated numbers of autophagosomes as indicated by punctate localization of GFP fluorescence. A, B, C, D, fixed tissue.

**Figure 7:**
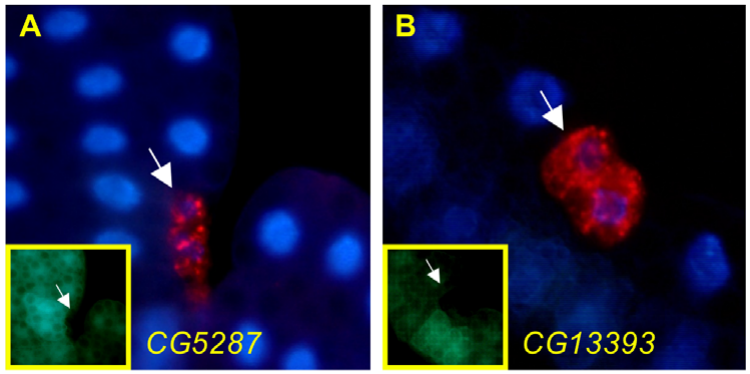
Disruption of a putative GlcNAc-1 phosphotransferase (*CG5287*) and the homolog of mammalian oligosaccharyl transferase (*CG13393*) cause increased LysoTracker staining. Mitotic mutant clones for P-element insertions in the genes *CG5287* (A) and *CG13393* (B) have elevated punctate LysoTracker staining. Inset: GFP expression identifies mutant clones (white arrows). Alleles used for mitotic clones: *CG5287^k10105^*, *CG13393^k12914^*.

One function of the ER translocon is to co-translationally import nascent transmembrane and secretory proteins into the ER for glycosylation and other processing steps via the signal recognition particle (SRP) and its receptor in the ER membrane. Once docked at the ER, the nascent polypeptide is co-translationally fed through the translocon. Because the translocon supports bidirectional traffic (unfolded proteins out of the ER, membrane and secretory pathway proteins in), we attempted to separate the import function by disrupting the SRP receptor and individual SRP subunits by RNAi. Knockdown of SRP subunits *Srp14*, *Srp54*, and *Srp68*, as well as the β subunit of the SRP receptor (*SrpRβ*) all caused marked increases in both LysoTracker staining (data not shown) and GFP-Atg8a punctae (Fig. 6A–D). In keeping with the importance of lysosomal degradation as a defense against defects in protein folding, mutation of a putative GlcNAc-1 phosphotransferase (*CG5287*) and the homolog of mammalian oligosaccharyl transferase (*CG13393*) cause an LT+ cellular phenotype (Fig. 7).

**Figure 8:**
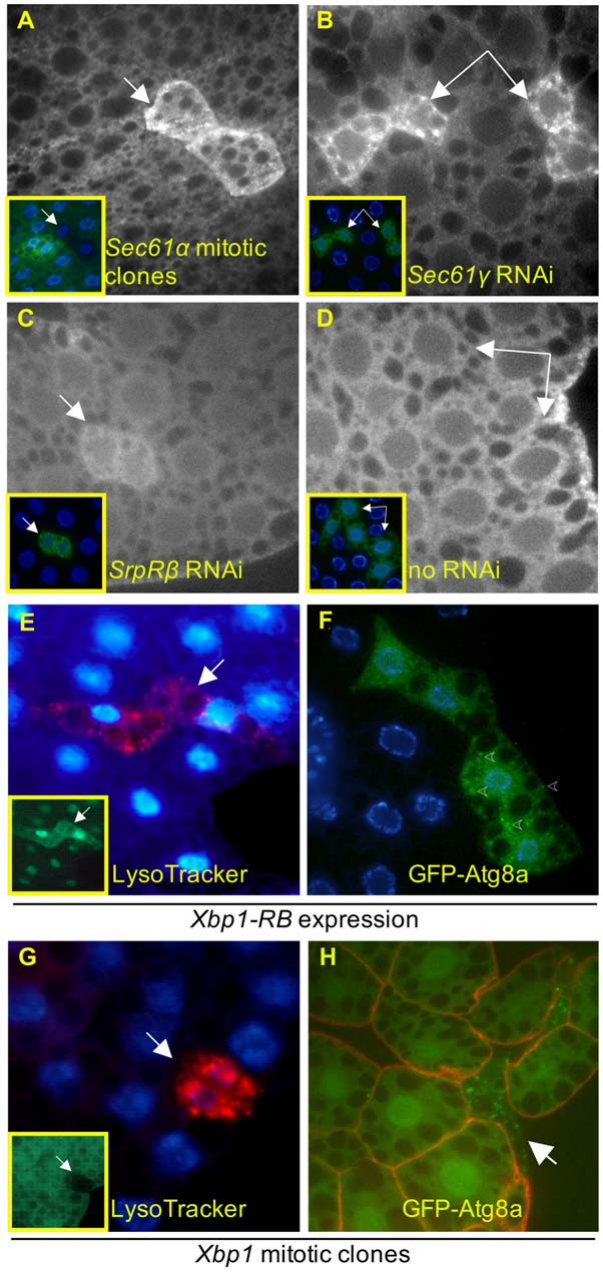
Involvement of the unfolded protein response in lysosomal expansion and autophagy. Fat body cells mutant for *Sec61α* (A) or expressing RNAi against *Sec61γ* or *SrpRβ* (B and C) and immunostained with antibody to Hsc70–3 have elevated levels of the protein relative to neighboring wild type cells, or cells expressing GFP alone (D). Inset panels and white arrows indicate cells of interest. Cells expressing UAS-driven constitutively active *Xbp1-RB* display punctate LysoTracker (E) and autophagosome formation (F; representative punctae indicated by arrowheads). Cells homozygous for a P-element insertion in *Xbp1* display both elevated LysoTracker (G) and autophagosome formation (H). Genotypes used for mitotic clone formation: *y w hsflp; Cg-GAL4 FRT42D UAS-GFP* (G) or *y w hsflp myr-dsRed FRT42D GFP-Atg8a* (H), *y w; FRT42D P{lacW}Xbp1^k13803^/SM6-TM6B*. A, B, C, D, F, H, fixed tissue; E, G, unfixed tissue.

Previous reports have shown that the UPR can activate autophagy, and it might be predicted that loss of ER translocon function could induce autophagy via the UPR. We thus investigated whether the *Sec61* and *SrpRβ* disruptions activated the UPR by immunostaining for *Hsc70–3*, the *Drosophila* homolog of the mammalian chaperone BiP, a commonly used indicator for UPR activation. Loss of *Sec61*α in mitotic mutant clones led to increased *Hsc70–3* staining, as did expression of RNAi against *Sec61γ* or *SrpRβ*, indicating activation of the UPR (Fig. 8).

To determine if UPR activation might play a causative role in autophagy induction in our system, we examined the role of *Xbp1*, a transcription factor that activates UPR genes in response to upstream signaling. Ectopic expression of *Xbp1-RB*, a constitutively active form of the protein, using the flip-out system induced both LysoTracker and GFP-Atg8a punctae (Fig. 8E–F), indicating that activation of this transcriptional UPR pathway is sufficient to trigger autophagy. Punctate LysoTracker and GFP-Atg8a were also observed in response to mitotic loss of *Xbp1* (Fig. 8G–H). In such cells, the absence of baseline *Xbp1* activity may lead to accumulation of unfolded proteins and activation of *Xbp1*-independent branches of UPR signaling. In support of this, we find that the Jun-kinase pathway is activated in *Sec61α* mutants and is sufficient on its own to induce autophagy (data not shown), indicating that multiple branches of UPR signaling may independently contribute to autophagy induction.

## Discussion

### Screen and Genomics

Our screen revealed a surprisingly large number of mutants, 15% of all tested, that increase lysosomal volume. In part, this reflects the selection of P-elements that disrupt essential genes, increasing the probability of impinging on critical pathways. The number of LT+ mutants also attests to the importance of the autophagy-lysosome pathway as a response to cellular damage or malfunction, since many of the LT+ mutations are in essential metabolic, biosynthetic, or quality control pathways. While many of the mutations likely cause significant cell stress or damage, the fact that the cells are still alive three or more days into development suggests that there may also be regulatory functions involved. It should also be noted that for some genes with two or more proximal lethal P-elements, only one was found to cause a LT+ phenotype (Table S1). This might be due to the hypomorphic nature of many P-element-associated mutations, or to the disruption of regulatory sequences of distant genes as opposed to proximal ones.

### tRNA Synthetases

Disruption of seryl- and threonyl-tRNA synthetases caused elevated LysoTracker staining, as did RNAi targeted against seven additional AARSs (Fig. 3 and data not shown). An excess of uncharged tRNAs resulting from decreased tRNA synthetase activity could induce autophagy by mimicking a nutrient-poor state, or by causing decreased fidelity of protein synthesis. However, the fact that tRNA disruption did not lead to GFP-Atg8a punctae argues against autophagy as the mechanism of action. Defects in *AlaRS* lead to misfolded proteins, accumulation of vacuoles, and cerebellar Purkinje cell loss in the *sticky* mutant mouse [19], and mutations in tRNA synthetase genes cause a number of heritable human diseases (reviewed in [20]). It is interesting to note that, while many of the known disease-specific mutations in human and mouse are limited to individual tRNA synthetases, the phenotype in our study appears to be more general. This could be because the sensitivity of our screen might identify mutations that would be subclinical, or that certain cell types are particularly sensitive to the loss of particular amino acids, such as those that act as neurotransmitters. Consistent with our findings, a recent report has shown that disruption of a wide range of AARS genes in *C. elegans* confers profound resistance to hypoxic stress through a reduction in hypoxia-induced cellular unfolded protein load [21]. While the implied mechanism of action was a reduction in protein synthesis, it is also possible that increased lysosomal protein clearance plays a role in the hypoxic resistance of AARS mutants.

### Mitochondrial Genes

The autophagy-lysosome pathway plays an essential role in destroying damaged or senescent mitochondria. Although the signals by which damaged mitochondria are recognized are unknown, recent results suggest that mitochondrial fission is critical to segregate ailing mitochondria for autophagic degradation [22]. Our screen identified several mitochondria-related genes, three of which—*Drp1*, *ab*, and *nmd—*have known or potential roles in mitochondrial fission and led to the formation of Atg8a-labelled autophagosomes. *Drp1* is a microtubule-associated GTPase that promotes mitochondrial fission (reviewed in [23]). Very little is known experimentally about *ab*, but disruption of the gene causes defects in mitochondrial morphology and subcellular localization (R. Cox, and A. Spradling, personal communication). Consistent with these results, we observe mitochondrial clumping in *ab* mutant cells. *Nmd* plays a role in mitochondrial aggregation in fly spermatid development [24], and its disruption in the FB causes both LT+ phenotype and GFP-Atg8a punctae.

There were also 4 LT+ mutants that play a role in the electron transport chain, including complex I (*CG12400*), complex III (*RISP*), and complex IV (*CG8885* and *cyt-c-p*). These mutants caused increased LysoTracker staining in the absence of discernable autophagosomes, suggesting they may lead to increased lysosomal activity through an autophagy-independent mechanism. Alternatively, they may cause an early or transient increase in autophagy which is not visible under our experimental conditions but persists in the form of increased lysosomal volume. This is supported by the fact that several of the mitochondrial mutants show reduced GFP-Atg8a levels, consistent with increased autophagic turnover of this fusion protein. It is also possible that the GFP-Atg8a and LysoTracker phenotypes are being caused by a block in the later steps of degradation, resulting in a pileup of autophagic intermediates which are formed and acidified, but fail to be destroyed. Those mitochondrial mutations that do induce autophagy may do so by causing energetic stress, by specific signaling such as the ROS that have been proposed to induce autophagy (reviewed in [25]), or by affecting the permeability transition that has also been proposed to play this role [22], [26].

### Autophagy, Protein Transit, and Quality Control

Autophagy was induced by disruption of the ER translocon, the SRP or its receptor, and a presumptive member of the oligosaccharyltransferase complex (OST) that catalyzes N-linked glycosylation of nascent polypeptides translocating into the ER [27]. In mammals, the OST complex is physically associated with ribosomes and with the ER translocon [28]. Previous reports show that loss of translocon function mitigates polyglutamine toxicity in the *Drosophila* eye [29], and that overexpression of *Sec61α* exacerbates polyglutamine toxicity [30]. The authors of that study hypothesize that retention of polyglutamine proteins in the ER prevents them from causing cytoplasmic toxicity, and that overexpression of *Sec61α* increases cytotoxic export. We would propose an alternative hypothesis that loss of translocon function induces autophagy, which plays a critical role in clearing neurotoxic aggregates [31]. In our system, overexpression of *Sec61α* was sufficient to suppress starvation-induced autophagy. *Sec61α*-mediated inhibition of autophagy provides a simple model for the accumulation of protein aggregates and cell death seen by Kanuka *et al*, namely that the *Sec61α* protein reduces autophagy through an unknown mechanism, interfering with degradation of toxic proteins.

It has been known for some time that ER stress activates autophagy and that autophagy provides a natural defense against diseases such as Huntington, Alzheimer's, and Parkinson's which are caused by toxic protein aggregates [4]—autophagy is required for long term survival of ER stress ([32] and reviewed in [33]). Many mechanisms have been proposed to account for the activation of autophagy by the UPR, including death associated protein kinase [34], PERK-eIF2-ασιγναλινγ [35], [36], IRE-1-JNK signaling [37], Ca^++^-CamKK-AMPK-TOR signaling [33], Ca^++^-dependent PKC activation [38], ER-resident 1,4,5-inositol trisphosphate receptors [39], and the chaperone GRP78/BiP [40]. It also appears that increased transcription of autophagy genes such as ATG5 and ATG12 may be involved [35], [36]. This may parallel our findings that constitutively active UPR transcription factor Xbp1 activates autophagy.

Existing evidence thus supports a wide range of distinct but not mutually exclusive routes from ER stress to autophagy. Given the absolute necessity for proper protein folding, secretion, and transmembrane insertion, the multi-faceted nature of the ER stress responses, and the role of autophagy in survival of transient ER failure, it is not surprising that multiple parallel ER-autophagy pathways might be in play. It remains to be shown if the many mechanisms of ER activation of autophagy are required in different contexts, are redundant systems, or reflect subtleties of the UPR that are not yet appreciated.

The screen we have described here is unique in a number of ways. Because of the large cell size and monolayer tissue architecture of larval fat body and the sophisticated recombination and gene expression tools generated by the *Drosophila* community, the mosaic approach in fat body allows for the visualization of cell biological phenotypes in live cells in the context of a fully developed wild type animal organ. This set of tools can thus be useful for a wide range of cellular phenotypes including cell size and shape, as well as other trafficking pathways or organellar compartments for which fluorescent dyes or markers exist such as mitochondria, ER, endosomes, etc. We therefore hope that our approach can be of use to other investigators seeking to screen for essential genes with observable cell biological phenotypes in an organismal, as opposed to a cell culture, context.

## Materials and Methods

### Fly Strains and Collections

The P-element insertion stocks were obtained from the Drosophila Genetic Resource Center of the Kyoto Institute of Technology (http://kyotofly.kit.jp/cgi-bin/stocks/data_search.cgi), or the Szeged Stock Center, Hungary (http://expbio.bio.u-szeged.hu/fly/index.php). The Kyoto stocks were generated and characterized by the BruinFly project [41] (http://www.bruinfly.ucla.edu/). UAS-RNAi stocks [42] were obtained from the Vienna Drosophila RNAi Center (http://stockcenter.vdrc.at/control/main) or the National Institute of Genetics of Japan in Shizuoka (http://www.shigen.nig.ac.jp/fly/nigfly/index.jsp). Other fly strains used: *w; uas-dSec61α^k155^/CyO* (kind gift of Masayaki Miura, University of Tokyo), *y w hsflp; Actin>CD2>GAL4, UAS-GFP* (“flip out”), *y w hsflp; Actin>CD2>GAL4, UAS-GFP-ATG8a*, *y w hsflp; r4-mCherry-Atg8a Act>CD2>GAL4 UAS-GFPnls/+, y w hsflp*; *UAS-2xeGFP FRT40A Fb-Gal4* (“40AGFP”), *y w hsflp; UAS-dsRed FRT40A fb-GAL4; UAS-GFP-Atg8a*, *y w hsflp; Cg-GAL4 UAS-GFP-Atg8a FRT42D UAS-myrRFP*, *y w; P{lacW}Xbp1^k13803^/CyO* (Bloomington Stock Center, #11104); *UAS-xbp1-RB* (kind gift of H.D. Ryoo [43]).

### EMS Mutagenesis and Screening Procedures

Mature isogenized males of the genotype *y w; FRT40A^iso1^* were fed overnight with 25 mM EMS in a 1.5% sucrose solution before a brief incubation in a clean vial and normal feeding. Males were crossed to virgin female *y w hsflp; sp/SM6b-TM6B*, and males of the genotype y w hsflp; FRT40A*/SM6-TM6B were retrieved. Individual males of the genotype FRT40A*/SM6-TM6B or multiple males of the genotype *FRT40A P/CyO* were crossed with 10–15 virgin females of the genotype *y w hsflp; UAS-2xeGFP FRT40A Fb-GAL4* or *y w hsflp; UAS-dsRed FRT40A fb-GAL4 UAS-GFP-Atg8a*. Eggs were collected in a normal food vial for 6–8 hours before a 1 hour heat shock at 37°C. Fat bodies were isolated from early third instar larvae and stained simultaneously with DAPI and 100 µM LysoTracker Red (L-7528, Molecular Probes) diluted in PBS. Fat bodies were then mounted in PBS on standard glass slides and coverslips and imaged on a Zeiss Axioskop 2 outfitted for epifluorescence. Mutant clones were identified by the lack of GFP and the close proximity of twin spot clones with two copies of the GFP expression cassette. All lines with a noticeable difference in size or LysoTracker staining in mutant clones *vs.* neighboring cells were noted. For phenotypic categorization, 3 clones per animal were visually inspected in a minimum of 3 animals, and multiple clones were imaged. For most crosses, 5 or more larvae were analyzed. For LT3 classification in Table S1, at least one clone per animal in multiple animals, but not necessarily all animals, was observed.

### Imaging Fixed Tissues and Antibody Staining

For confocal imaging of fluorescent ATG8 fusion proteins and antibody staining, larvae were everted and fixed in 4% formaldehyde in PBS at RT for 90 minutes before washing with PBS/0.01 triton X-100 (PBS-TX), staining with DAPI, washing in PBS, and dissection and mounting in FluoroGuard (Bio-Rad). Anti-Hsc70-3 guinea pig polyclonal antibody (a kind gift of H.D. Ryoo) was diluted 1∶200 in PBS-TX. Everted larvae were incubated in primary antibody for 2 hours at room temperature or overnight at 4°C, washed 3 times in PBS-TX, incubated in Texas Red-conjugated anti-GP secondary antibody for 1–2 hours at RT, washed and stained in DAPI and mounted as above, and imaged on a Zeiss Axioskop2 microscope (Plan-Apochromat 63×1.40 NA objective) equipped with a CARV spinning disc confocal system and a Hamamatsu ORCA-ER digital camera.

### Flipout Expression of UAS-driven Genetic Constructs

UAS expression stocks were crossed to virgins of y w hsflp; Actin>CD2>GAL4, UAS-GFP, y w hsflp; Actin>CD2>GAL4, UAS-GFP-ATG8a or y w hsflp; r4-mCherry-Atg8a Act>CD2>GAL4 UAS-GFPnls/+, and incubated at 25°C for 3–4 days. Leaky expression at 25°C of the heat-shock inducible FLP leads to stochastic “flip out” activation of GAL4 expression [44] in a minority of fat body cells, activating UAS-driven expression of GFP and the inserted RNAi, allowing assessment of these cells' level of lysosomal and autophagic activity in otherwise normal animals. Fat bodies were either stained unfixed with LysoTracker and DAPI, or were fixed and imaged as noted.

### Gene Ontology

The GO Term Mapper (http://go.princeton.edu/cgi-bin/GOTermMapper) was used to bin individual GO categories into more general parent or “slim” terms to facilitate a broader analysis of functional categories. FlyBase IDs for the LysoTracker-positive and -negative groups with duplications eliminated were uploaded as text files to the GO Term Mapper and run against the FlyBase gene association file. Each group of genes was run separately in all three GO databases, and were compared to one another and to the overall fly genome using Microsoft Excel.

## Supporting Information

Table S1 Full listing and phenotype data for all strains tested. Table lists all P-element insertion stocks and RNAi insertion stocks tested, from a total of four stock centers. Phenotype designations are as follows: for RNAi expression experiments LysoTracker positive phenotypes were listed simply as LT+. For FRT40A mitotic clones, LT+ phenotypes were classified as LT1 (intense punctate staining); LT2 (mild to moderate, with some punctae); LT3 (mild or intermittent phenotype, including diffuse as opposed to punctate staining); S (small cell size without LysoTracker phenotype); and L (apparent cell lethal). Type (mitotic or RNAi) and source of constructs are listed, and can be sorted by any field. Gene disruption assignments for P-element insertions were downloaded from previously published descriptions and initial characterizations of the P-element collections: http://www.genetics.org/cgi/content/full/163/1/195/DC1 [45] and http://www.bruinfly.ucla.edu/subsets.php?id=3. Where two genes were potentially disrupted, both are listed. Additional information was obtained by personal communication from the curators of the BruinFly project and from FlyBase release 5.15. For BruinFly stocks, the primary ID listed is the Kyoto Stock Center ID; BruinFly ID is listed separately. Insertion site refers to the genomic location of the P-element insertion or the chromosome of insertion of the UAS-RNAi insert, as appropriate.
(0.47 MB XLS)
